# Exploring personalized structural connectomics for moderate to severe traumatic brain injury

**DOI:** 10.1162/netn_a_00277

**Published:** 2023-01-01

**Authors:** Phoebe Imms, Adam Clemente, Evelyn Deutscher, Ahmed M. Radwan, Hamed Akhlaghi, Paul Beech, Peter H. Wilson, Andrei Irimia, Govinda Poudel, Juan F. Domínguez Duque, Karen Caeyenberghs

**Affiliations:** Leonard Davis School of Gerontology, University of Southern California, Los Angeles, CA, USA; Healthy Brain and Mind Research Centre, School of Behavioural, Health, and Human Sciences, Faculty of Health Sciences, Australian Catholic University, Fitzroy, Victoria, Australia; Cognitive Neuroscience Unit, School of Psychology, Faculty of Health, Deakin University, Burwood, Victoria, Australia; KU Leuven, Department of Imaging and Pathology, Translational MRI, Leuven, Belgium; Emergency Department, St. Vincent’s Hospital (Melbourne), Faculty of Health, Deakin University, Melbourne, Victoria, Australia; Department of Radiology and Nuclear Medicine, The Alfred Hospital, Melbourne, Victoria, Australia; Corwin D. Denney Research Center, Department of Biomedical Engineering, Viterbi School of Engineering, University of Southern California, Los Angeles, CA, USA; Department of Quantitative and Computational Biology, Dana and David Dornsife College of Arts and Sciences, University of Southern California, Los Angeles, CA, USA; Mary MacKillop Institute for Health Research, Australian Catholic University, Melbourne, Victoria, Australia

**Keywords:** Traumatic brain injury, Structural connectomics, Graph theory, Personalized medicine, Personalized connectomics, Lesion filling

## Abstract

Graph theoretical analysis of the structural connectome has been employed successfully to characterize brain network alterations in patients with traumatic brain injury (TBI). However, heterogeneity in neuropathology is a well-known issue in the TBI population, such that group comparisons of patients against controls are confounded by within-group variability. Recently, novel single-subject profiling approaches have been developed to capture inter-patient heterogeneity. We present a personalized connectomics approach that examines structural brain alterations in five chronic patients with moderate to severe TBI who underwent anatomical and diffusion magnetic resonance imaging. We generated individualized profiles of lesion characteristics and network measures (including personalized graph metric GraphMe plots, and nodal and edge-based brain network alterations) and compared them against healthy reference cases (*N* = 12) to assess brain damage qualitatively and quantitatively at the individual level. Our findings revealed alterations of brain networks with high variability between patients. With validation and comparison to stratified, normative healthy control comparison cohorts, this approach could be used by clinicians to formulate a neuroscience-guided integrative rehabilitation program for TBI patients, and for designing personalized rehabilitation protocols based on their unique lesion load and connectome.

## Author Summary

Single-subject profiling captures heterogeneity of the structural connectome to characterize brain network alterations in patients with traumatic brain injury (TBI). We profile individual patients based on their unique brain graphs in comparison to healthy controls, to provide insights into brain network disruption otherwise obscured by group-level approaches. Our implementation extends current methods by analyzing TBI structural profiles when automatic sub/cortical segmentation or parcellation of MRIs fail in the presence of lesions. Our approach highlights the translational potential for single-subject network analyses in the study of brain injury. Personalized connectome profiling provides a novel user-friendly framework for leveraging graph metrics to benefit the individual patient, by characterizing network-level brain alterations with potential prognostic relevance.

## INTRODUCTION

Moderate to severe traumatic brain injury (TBI) can result in diverse focal lesions and white matter pathology. The locations of these lesions greatly contribute to functional outcomes following TBI, whereby cognitive functions that rely on broadly distributed circuits in the brain are affected due to disruptions to axonal pathways and cortical structures (Bressler & Menon, 2010; Catani & Ffytche, 2005; Hampshire et al., 2016). In TBI patients, diffusion-weighted MRI (dMRI) studies have shown altered topological properties of structural brain networks, as indexed by graph metrics at the group level (Caeyenberghs et al., 2014; Kim et al., 2014; Raizman et al., 2020; van der Horn et al., 2017). In our recent meta-analysis (Imms et al., 2019), we found that only two of 14 graph metrics (characteristic path length and normalized clustering coefficient) showed significant differences in TBI patients compared with controls, reflecting the heterogeneous nature of TBI patients. This heterogeneity, including complex structural profiles, variation in lesion location, severity, response to treatment, as well as varied secondary injury trajectories, poses a challenge for the prediction of functional and cognitive symptoms of TBI patients. As a result, there is growing impetus for subject-tailored approaches that enable injury characterization and treatment planning (Irimia, Chambers, et al., 2012; Irimia, Wang, et al., 2012; Jolly et al., 2021).

Recent studies have addressed heterogeneity in clinical cohorts by performing individualized analyses of dMRI-derived fractional anisotropy (FA), T1-derived cortical thickness, and streamline counts (Attyé et al., 2020; Jolly et al., 2021; Lv et al., 2020) at the level of white matter tracts or gray matter regions, respectively. For example, Lv et al. (2020) found no group consensus in anatomic locations of lower FA and reduced cortical thickness in schizophrenia patients, and as such group-level FA and cortical thickness maps were not representative of individuals. To date, however, few studies have analyzed brain networks at the level of individual patients, an approach known as *personalized connectomics* (Irimia, Wang, et al., 2012).

Pioneered by Irimia, Chambers, et al. (2012), personalized connectomics enables the use of an individual’s brain network as a “fingerprint” of brain network topology (Sanz Leon et al., 2013; Schirner, Rothmeier, Jirsa, McIntosh, & Ritter, 2015). Personalized connectomics allows the visualization of individual white matter atrophy profiles (as indexed by dMRI-inferred streamline counts) using circular plots and considering patients’ scores relative to a healthy cohort. These individualized graphs can be used by clinicians to develop personalized rehabilitation programs, by detailing network-level abnormalities that may indicate specific cognitive deficits following injury (Irimia, Chambers, et al., 2012). No study to date has examined TBI patients’ network alterations using graph metrics, whereby a literature-driven selection of graph metrics that summarize segregation, integration, and centrality are represented for individual patients (Rubinov & Sporns, 2010). Since graph metrics were recently shown to have prognostic potential (Roine et al., 2022; van der Horn et al., 2017), this type of approach could provide valuable information to clinicians, leading to neuroimaging-guided strategies to improve functional outcomes of TBI patients. However, personalized connectomics in moderate to severe TBI cohorts with diverse brain injuries pose a serious technical challenge, as the available tools for MRI processing to generate connectomes fail in such conditions (King et al., 2020).

The present study introduces personalized measurement and analysis of individual connectomic profiles in five chronic moderate to severe TBI patients with varying lesion loads, mechanisms of injury, age at injury, and burden of neural/cognitive symptoms. Our implementation extends current methods by addressing the long-standing and prominent challenge of analyzing TBI structural profiles when automatic sub/cortical segmentation or parcellation of MRIs fail in the presence of lesions (King et al., 2020). Significantly, this problem is addressed here by synergizing connectomic analysis with *virtual brain repair*, where the lesion is replaced by healthy-looking tissue in the T1-weighted images (lesion inpainting). The capabilities of our implementation of personalized connectomics in TBI include the following: (a) *lesion masking* undertaken in a semiautomated manner from anatomical T1 MRI scans to identify the affected brain regions in individual patients; (b) the use of the recently developed Virtual Brain Grafting (VBG) toolbox to overcome the challenges of segmentation and parcellation of focal lesions using lesion inpainting (Radwan et al., 2021); (c) graphical representation of the structural connectome using innovative tools for graph metric profiling (GraphMe plots) to delineate subject-specific changes in brain network integration, segregation, and centrality; and (d) regional assessment of network hub regions and edge alterations in individual TBI cases. Together, these innovative solutions overcome major, long-standing methodological impediments in the field of macroscale TBI profiling. Our implementation is the first to allow the comprehensive generation of lesion-aware connectomic profiles, thus moving closer to the crucial aim of quantifying brain network alterations in the individual TBI patient.

## METHODS

### Participants

Patients with chronic moderate to severe TBI were recruited from St. Vincent’s Hospital in Melbourne. The definition of moderate to severe TBI was based on (a) a Glasgow Coma Scale score between 3 and 12 at the time of hospital admission (Teasdale & Jennett, 1974); (b) loss of consciousness of at least 30 min; (c) post-traumatic amnesia of at least 24 hr (Rabinowitz & Levin, 2014); and (d) positive findings of gross injury on MRIs as per evaluation by a neuroradiologist (PB). Patients who met the following inclusion criteria were contacted to take part in the study: (a) between 18 and 65 years of age; (b) no history of head injury prior to the TBI for which they were included in this study; (c) fluency in English; (d) no history of psychiatric illness prior to the TBI; and (e) no contraindications for MRI. Ten moderate to severe TBI patients who had sustained closed head injuries due to sports or motor vehicle accidents more than 6 months prior to the study were recruited. Informed written consent was obtained from each subject in accordance with the Declaration of Helsinki. Because of time constraints during scanning, dMRI were not acquired from four TBI patients, who were subsequently removed from further analysis (see Table 1). One participant was removed from personalized connectome construction because of excess movement in the scanner during dMRI, which caused a severe motion artifact (see Supplementary Material 1 in the Supporting Information for their quality assessment). For the reference group, 12 healthy controls were recruited from the general population using flyers and the snowball method. Ethical approval was granted by the St. Vincent’s Hospital Melbourne ethics committee for human research (Project No. 250/17).

**Table 1.** Participant demographics and injury characteristics

**ID**	**Age** ^**a**^	**Sex**	**TSI** ^**b**^	**Mechanism**	**Pathology (at time of study)** ^**c**^	**DAI** ^**d**^
HC	35.7 ± 11.4	M = 4 F = 8	–	–	No incidental or age-related findings, other than small deep white matter T2 hyperintensities (within normal limits for age).	–
TBI1	40s	M	21y	Vehicle accident	Modest encephalomalacia in the (R) precentral gyrus.	0
TBI2	40s	M	15y	Vehicle accident	Severe encephalomalacia involving both ant. F and inf. F lobes, (R) T lobe and (R) parietotemporal region extending to the (R) post. F lobe. Focal T1 hypointensities in the anteromedial portion of the (L) thalamus. Encephalomalacia and T1 hypointensity on the ant. body and genu of the corpus callosum.	2
TBI3	40s	F	3y	Fall	Bilateral ant. and inf. F encephalomalacia, (R) greater than (L), and (R) ant. T encephalomalacia. Small deep white matter T2 hyperintensities med. (R) P lobe, likely associated with non-hemorrhagic oedema. Small focal T1 hypointensity in the ant. body of the corpus callosum.	2
TBI4	30s	F	15y	Fall	Bilateral inf. F and (L) ant. T encephalomalacia. Modest encephalomalacia in the (L) sup. F gyrus. (R) F ventriculostomy with underlying ventricular drain tract.	0/1
TBI5	50s	M	18y	Vehicle accident	Two small (<2 mm^3^) deep white matter T2 hyperintensities in the (R) P lobe (within normal limits for age).	0
TBI6	30s	F	5y	Fall	Small T1 hypointensity in the splenium of corpus callosum. Approx. 6 scattered punctate T2 hyperintensities in both cerebral hemispheres.	2

^a^ Age: Shown in 10-year age bracket to minimize identifiable information, HC age is in mean ± standard deviation.

^b^ TSI: Time since injury.

^c^ Abbreviations: (R) = right, (L) = left, ant. = anterior, post. = posterior, inf. = inferior, mid. = middle, med. = medial, sup. = superior, F = frontal, P = parietal, O = occipital, T = temporal.

^d^ Grading of diffuse axonal injury (DAI) occurred according to Adams et al. (1982); a grade of 0 indicates no confirmed DAI present; 1 indicates DAI present in white matter of cerebral hemispheres, corpus callosum, brain stem, cerebellum; 2 indicates there is also a focal lesion in corpus callosum; and 3 identifies an additional lesion in dorsolateral quadrants of brain stem.

### Data Acquisition

MRI scans were acquired at the Royal Children’s Hospital using a 3T Siemens PRISMA with a 64-channel head coil. dMRI data were acquired using a single-shot echo planar imaging sequence (twice-reinforced spin echo, multiband acceleration factor of 2, 70 contiguous sagittal slices) and a high angular resolution diffusion imaging (HARDI) gradient scheme with 66 noncollinear gradient directions (total acquisition time [TA] = 6:17 min, *b* = 3,000 s/mm^2^, field of view [FOV] = 260 mm^2^, voxel size = 2.3 mm isotropic, repetition time [TR] = 3,500 ms, echo time [TE] = 67 ms, seven volumes with *b* = 0, two reverse phase-encoded volumes with *b* = 0, *b* being the constant of diffusion weighting). T1-weighted MRIs were also acquired using a magnetization-prepared rapid acquisition gradient-echo (TA = 5:48 min, 208 contiguous slices, FOV = 256 mm^2^, voxel size = 0.8 mm isotropic, TR = 2,100 ms, TE = 2.22 ms, flip angle = 8°).

### Lesion Masking

Manual lesion delineation for computation of lesion load and for improvement of anatomical segmentation was performed by an assessor (ED), who was trained in lesion identification by neuroradiologist (PB). Lesions were drawn in the T1 native space using FSLeyes version 0.27.3 in FSL version 6.0.1 (https://fsl.fmrib.ox.ac.uk/fsl/fslwiki). An in-house systematic search method and lesion identification protocol was implemented by JD, KC, ED, and PB. Abnormalities resulting in tissue loss, such as regions of encephalomalacia and damage from surgical drainage tracts, were included in binarized lesion masks. Enlarged ventricles and hyperintensities often occurring in proximity to the skull (e.g., from surgical craniotomies) were not included in the lesion masks. Lesion load was computed (in cm^3^) as the total volume of the binary lesion masks in FSL. Grading of diffuse axonal injury (DAI) was performed by expert raters PB and ED (Table 1; Adams, Graham, Murray, & Scott, 1982).

### Personalized Connectome Construction

**Figure 1.**
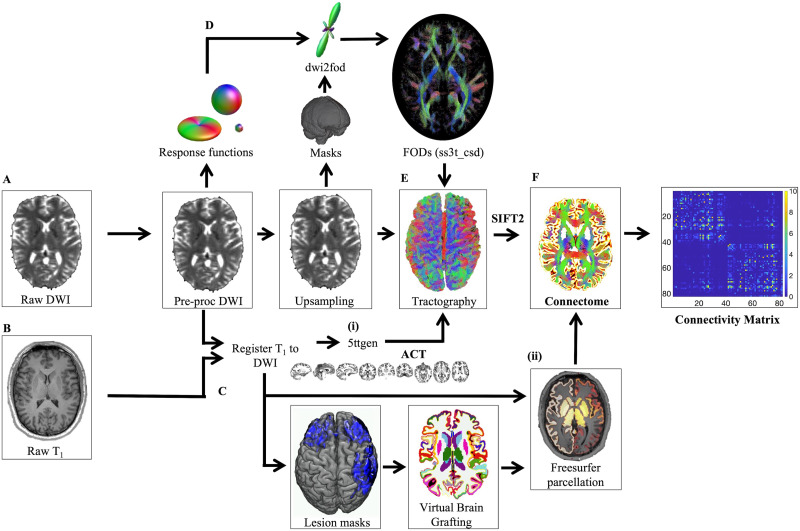
Overview of the processing pipeline for connectome mapping. (A) In the raw diffusion images, noise (Cordero-Grande et al., 2019; Veraart et al., 2016), Gibbs ringing artifacts (Kellner et al., 2016), as well as distortions induced by motion, eddy current artifacts, and EPI/susceptibility distortions were detected and corrected (Andersson et al., 2003; Andersson & Sotiropoulos, 2016). (B) Concurrently, T1 volumes were registered to diffusion volumes. The advanced normalization tools package (ANTS; Avants et al., 2009) was used to remove non-brain structures from the T1-weighted images for white matter extraction (Zhang et al., 2011). FSL FLIRT (Jenkinson et al., 2002; Jenkinson & Smith, 2001) was used to perform the boundary-based registration between brain-extracted anatomical and diffusion images. Registered images are provided to (i) 5ttgen (brain extracted), to create priors for anatomically constrained tractography (ACT), and (ii) FreeSurfer (non-brain extracted), to parcellate the nodes for the connectome analysis. All subcortical gray matter structures were segmented (Fischl et al., 2002); image intensity normalized (Sled et al., 1998); pial surfaces and the gray-white matter boundaries estimated (Dale et al., 1999); and the entire brain “inflated” to smooth the gyri and sulci (Fischl et al., 1999). (C) Lesion masks of subjects who failed the quality assessment after FreeSurfer parcellation were provided along with the T1 image to VBG. (D) Average response functions for white matter, gray matter, and cerebrospinal fluid were estimated from the dMRI data using an automated unsupervised approach (Dhollander et al., 2019; Dhollander et al., 2016). Preprocessed data were upsampled to a voxel size of 1.3 mm^3^ to assume higher spatial resolution for image registration before binary masks were created. Fiber orientation distributions (FODs) were estimated from the group average response functions on upsampled images, and corrected for intensity inhomogeneity and global intensity level differences (Raffelt et al., 2017). (E) Anatomically constrained tractography (ACT) was performed using the FODs from panel D and the 5ttgen images from panel B(i). The FOD cutoff threshold, step size, and angle were determined to attain a reasonable trade-off between false negatives and false positives (seed points = dynamic; maximum length = 250 mm; minimum length = 5 mm; step size = 1.25; angle = 45°; FOD amplitude = 0.08). Spherically informed filtering of tractograms (SIFT2) is applied to make the weight of the streamlines proportional to the underlying fiber orientation distribution. (F) The connectome is created using the FreeSurfer parcellation and the sifted tractogram.

Our connectome processing pipeline is showcased in Figure 1 and in Supplementary Material 2 in the Supporting Information, and in our previous publication (Imms et al., 2021). Our personalized connectomics implementation performs state-of-the-art, single-subject analyses of structural MRI scans. Briefly, raw dMRI data were processed using MRtrix3Tissue (v5.2.8; https://3tissue.github.io), a fork of MRtrix3 (Tournier et al., 2019). White matter fiber orientation distributions were estimated using single-shell 3-tissue constrained spherical deconvolution (SS3T-CSD; Dhollander & Connelly, 2016; Khan et al., 2020). Whole-brain, anatomically constrained tractography (ACT) was performed (Smith, Tournier, Calamante, & Connelly, 2012) and 22 million streamlines were generated per subject (Yeh, Smith, Liang, Calamante, & Connelly, 2018). The spherically informed filtering of tractograms (SIFT2) algorithm was applied to match the fiber density of the reconstructed streamlines to that of the underlying white matter structures (Smith, Tournier, Calamante, & Connelly, 2015a, 2015b; Yeh et al., 2018). Thus, edges encode filtered streamlines count. Compared with tractograms reconstructed simply by the number of streamlines, SIFT2 modulates the weight of individual streamlines so that the tractogram is aligned with the underlying image data (Smith, Calamante, et al., 2020; Smith, Raffelt, et al., 2020; Smith et al., 2015a, 2015b). SIFT2 has high reproducibility (Girard et al., 2020; Koch et al., 2022) and increases the biological interpretability of the white matter tracts estimated (Frigo et al., 2020; McColgan et al., 2018). McColgan et al. (2018) also found that compared with unfiltered tractograms, SIFT2 improved the detection of group differences and lead to stronger clinical correlations.

*T*_1_ anatomical MRIs were parcellated into 84 regions of the Desikan-Killiany atlas (Desikan et al., 2006) using FreeSurfer’s *recon-all* function (v6.0; https://surfer.nmr.mgh.harvard.edu/; Fischl & Dale, 2000). Two patients (TBI3 and TBI4) had significant segmentation failures due to gross pathology, and were therefore processed utilizing VBG v0.37 (Radwan et al., 2021). Rather than lesion masking and manual editing, which are subjective and time-consuming, VBG automatically fills uni- and bilateral brain lesions using synthetic healthy donor tissue to permit or to improve segmentation. To illustrate the performance of VBG in TBI, we included a report on VBG outcome for patient TBI2, who was excluded from personalized connectomics because of movement during HARDI acquisition but otherwise had a quality control compliant T1-weighted volume (see Supplementary Material 3 in the Supporting Information). Given that VBG artificially reconstructs lesioned nodes, part of our quality control also included ensuring streamlines were not aberrantly assigned to these nodes. Connectivity matrices were generated using edge weights from SIFT2 and nodes defined as brain regions from FreeSurfer and VBG.

Robustness testing was performed on this exact pipeline (except VBG) in our recent publication (Imms et al., 2021), where a series of control analyses were performed assessing (a) atlas/parcellation schemes, (b) streamline normalization to regional volume, and (c) weight-to-length remapping procedures. The results of control analyses indicated optimal performance using (a) the Desikan-Killiany atlas (Desikan et al., 2006), (b) streamline weighting variant to the volume of each node (Smith, Calamante, et al., 2020), and (c) the use of standard remapping procedures (Rubinov & Sporns, 2010; Seguin, van den Heuvel, & Zalesky, 2018).

Global network properties were quantified in terms of strength, global efficiency, characteristic path length, navigation efficiency, average local efficiency, clustering coefficient, normalized clustering coefficient, and average betweenness centrality (Table 2) using the Brain Connectivity Toolbox (Rubinov & Sporns, 2010). These graph metrics were chosen from all available metrics as the most clinically informative/intuitive according to our meta-analysis (Imms et al., 2019), and graph theory studies in TBI (Jolly, Scott, Sharp, & Hampshire, 2020; Raizman et al., 2020; S. Wang et al., 2021) published after our meta-analysis. Specifically, we selected (a) normalized clustering coefficient and characteristic path length, which showed *robust* alterations in TBI patients compared with healthy controls (Imms et al., 2019); (b) global efficiency, betweenness centrality, strength, average local efficiency, and clustering coefficient, which showed significant differences with healthy controls and correlations with cognitive outcome measures in TBI patients (as shown in Table 2); and (c) navigation efficiency as a biologically meaningful measure of brain network communication and proxy for cognition (Imms et al., 2021; Seguin et al., 2019; Seguin et al., 2020).

**Table 2.** Graph metric descriptions and interpretations

**Graph metric**	**Description**	**Higher values mean …**	**Previous studies (*Adult msTBI*^*a*^)**	**Related to …**
***Integration***				
Characteristic path length	The shortest path is the fastest and most direct communication pathway between two network nodes. Characteristic path length is defined as the average shortest path length between all node pairs in a network (Watts & Strogatz, 1998).	A higher characteristic path length indicates that the fastest communication pathways between regions are, on average, longer and **less** efficient.	**Higher** characteristic path length (Caeyenberghs et al., 2014; Hellyer et al., 2015; Kim et al., 2014; Pandit et al., 2013; S. Wang et al., 2021).	Verbal learning, executive dysfunction (Kim et al., 2014). Intelligence, working memory span (Königs et al., 2017). Cognitive flexibility and information processing (Hellyer et al., 2015).
Global efficiency	The inverse average shortest path efficiency between all possible pairs of nodes in a network, where efficiency is computed as the inverse of the shortest path length (Latora & Marchiori, 2001).	A higher global efficiency indicates a **greater** capacity for efficient integration of information (in parallel) across the network.	**Lower** global efficiency (Caeyenberghs et al., 2014; Kuceyeski et al., 2016; Pandit et al., 2013; S. Wang et al., 2021).	Switching task/attention (Caeyenberghs et al., 2014).
Navigation efficiency	Navigation paths use a decentralized and geometrically greedy heuristic (Boguna et al., 2009). Navigation efficiency is defined as the average navigation path efficiency between all possible pairs of nodes in a network (Seguin et al., 2018).	Higher navigation efficiency indicates **greater** capacity for efficient integration of information across the network.	Not yet investigated, but **lower** navigation efficiency observed in stroke patients (X. Wang et al., 2019).	
***Segregation***				
Clustering coefficient	The number of existing connections between the neighbors of a node, divided by all the possible connections, calculated for each node individually and averaged across the entire network (Watts & Strogatz, 1998).	A higher average clustering coefficient implies that a greater proportion of connections are made between node neighbors, compared with the connections possible, and indicates more clustered connectivity around individual nodes.	**Lower** clustering coefficient (Hellyer et al., 2015; Raizman et al., 2020).	Cognitive flexibility and information processing (Hellyer et al., 2015).
Normalized clustering coefficient	Clustering coefficient of the network normalized to a random network.	Higher normalized clustering indicates **higher** local specialization, with a value of 1 being equivalent to a random network. If greater than 1, the network has greater than random clustering. There may be a point of diminishing returns, where greater local specialization comes at the **cost** of communication efficiency.	**Higher** normalized clustering^b^ (Caeyenberghs, Leemans, De Decker, et al., 2012; Verhelst et al., 2018).	Processing speed (van der Horn et al., 2017).
Local efficiency	The local efficiency is the average of inverse shortest path length in a local area. Mean local efficiency is the efficiency of each node in the network averaged over the total number of nodes (Latora & Marchiori, 2001).	A higher local efficiency means **greater** capacity for integration between the immediate neighbors of a given node.	**Higher** local efficiency (Jolly et al., 2020); **and/or lower** local efficiency (Caeyenberghs, Leemans, De Decker, et al., 2012).^b^	Reasoning, working memory (Jolly et al., 2020).
***Centrality***				
Strength	The strength of a node is the sum of the weights of its edges. Mean strength is the average of all the normalized strength values across each node of the network.	A higher strength indicates a **greater** average edge weight for each node.	**Lower** strength (Raizman et al., 2020).	
Betweenness centrality	The proportion of shortest paths that pass through node *i* between its neighboring nodes, calculated for each node and averaged across the network (Freeman, 1978).	Higher betweenness centrality means the node lies on more shortest paths in the network, and thus the node is **more central** and important to the network. A high network / average betweenness centrality indicates a high number of nodes that are central to shortest paths.	**Higher** betweenness centrality (Caeyenberghs, Leemans, De Decker, et al., 2012).^b^	Associative memory (Fagerholm et al., 2015).

^a^ msTBI: Moderate to severe traumatic brain injury.

^b^ This study is of young adults *and* children with TBI.

### Brain Network Profiles

Graph metric spiderplots (GraphMe plots) show results for each TBI patient in a concise and intuitive manner relative to mean scores from the healthy controls with 95% confidence intervals (see Supplementary Material 4 in the Supporting Information). Selected graph metrics (characteristic path length, normalized clustering coefficient, and betweenness centrality—Table 2) were inverted (1/*x*) to facilitate interpretation (so that *higher* scores on any graph metric denote *better* brain network structure). Correction for differences in brain sizes was performed by dividing each graph metric by the inverse of their total intracranial volume. Important to note, node area-size normalization was not performed, as we have previously found that variance in node size is a feature of interest in the human brain network when using edge weights based on SIFT2, which lead to stronger correlations with cognition (Imms et al., 2021; Smith, Raffelt, et al., 2020). Graph metrics of individual patients were categorized as follows: *normal* (if the scores/metrics fell within the 95% confidence interval); *supra-normal* (higher than the 95% confidence interval); or *infra-normal* (lower than the 95% confidence interval) (Lv et al., 2020).

### Regional Brain Network Analyses

**Figure 2.**
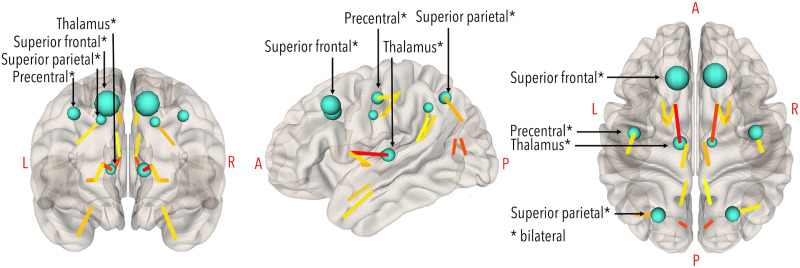
Healthy control hubs (top 10% of nodes with highest betweenness centrality), in teal. Larger nodes represent higher values. Hubs (bilaterally) were the superior frontal gyrus (BC_left_ = 1,493; BC_right_ = 1,533), superior parietal gyrus (BC_left_ = 610; BC_right_ = 665), precentral gyrus (BC_left_ = 588; BC_right_ = 616), and thalamus (BC_left_ = 336; BC_right_ = 346). The strongest edges (0.5th percentile) are colored by strength (yellow = weaker; red = stronger). Visualization in NeuroMArVL (https://immersive.erc.monash.edu/neuromarvl/).

**Figure 3.**
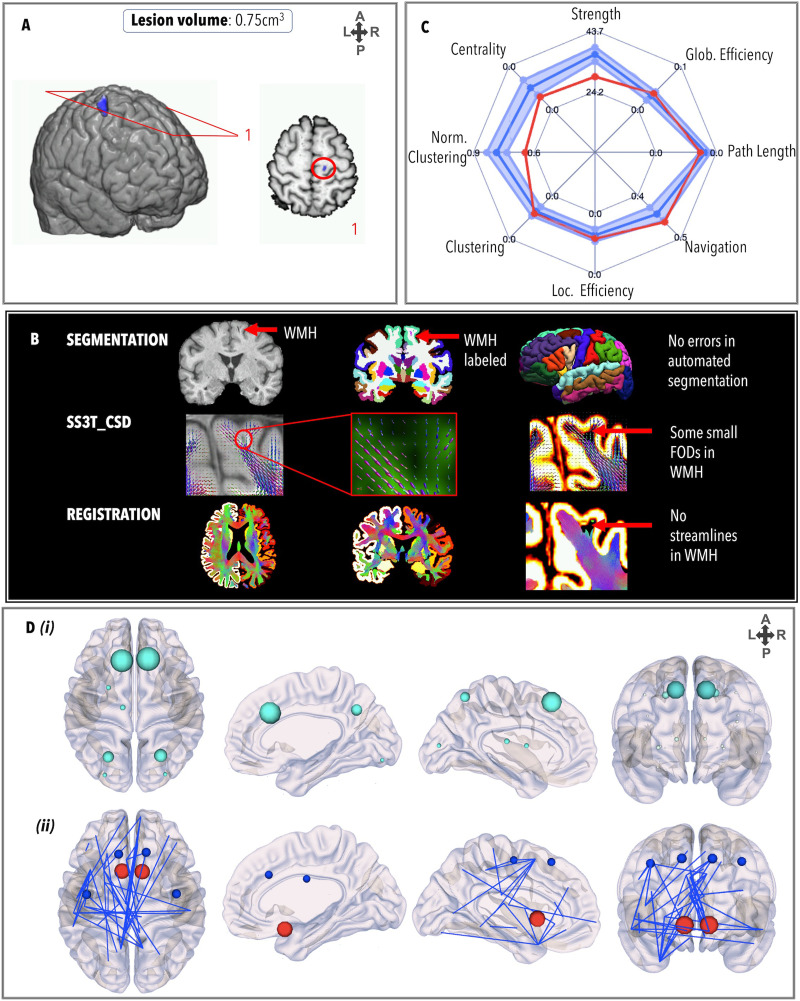
Personalized connectome profile for TBI1 including (A) lesion profile; (B) quality assessment; (C) radar plot showing the patient’s personalized connectome profile (red indicates patient’s scores, dark blue indicates healthy control average and the 95% CI is represented by the blue shade); and (D) (i) hub nodes (size indicates betweenness centrality value) and (ii) regional analysis (blue = edges/nodes lower than the healthy control average; red = edges/nodes stronger than the healthy control average).

**Figure 4.**
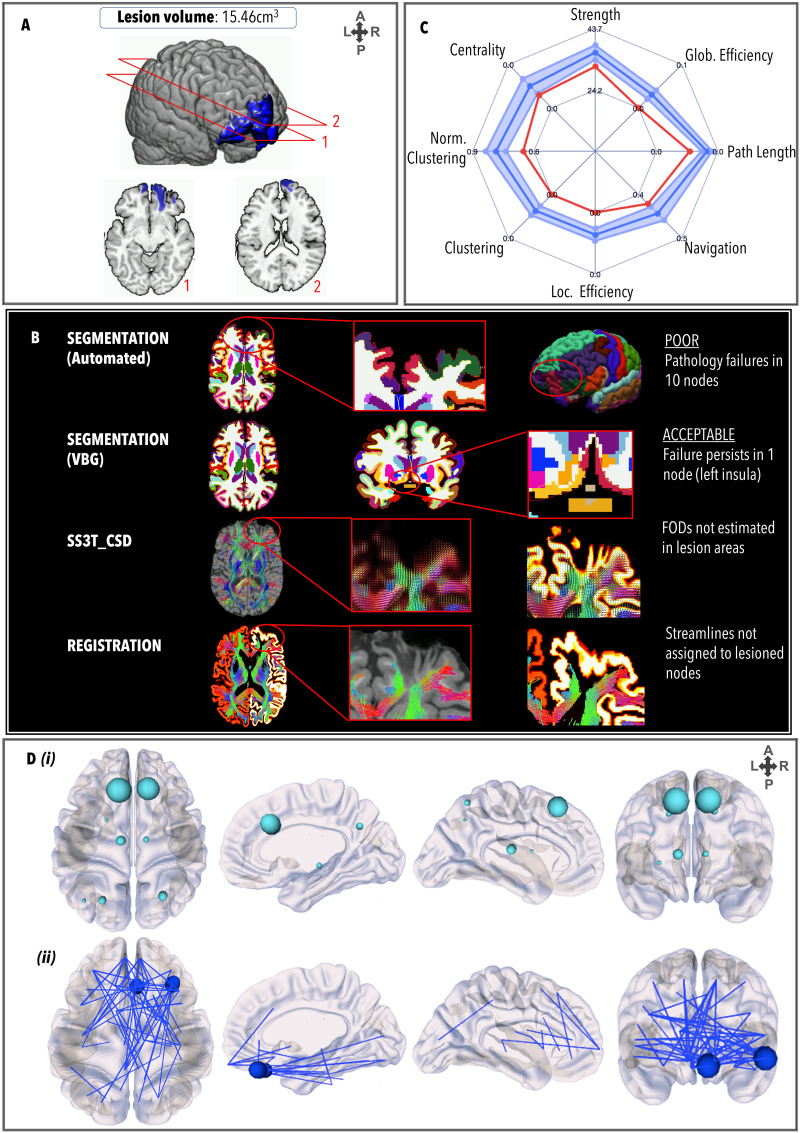
Same as Figure 3, for TBI3.

**Figure 5.**
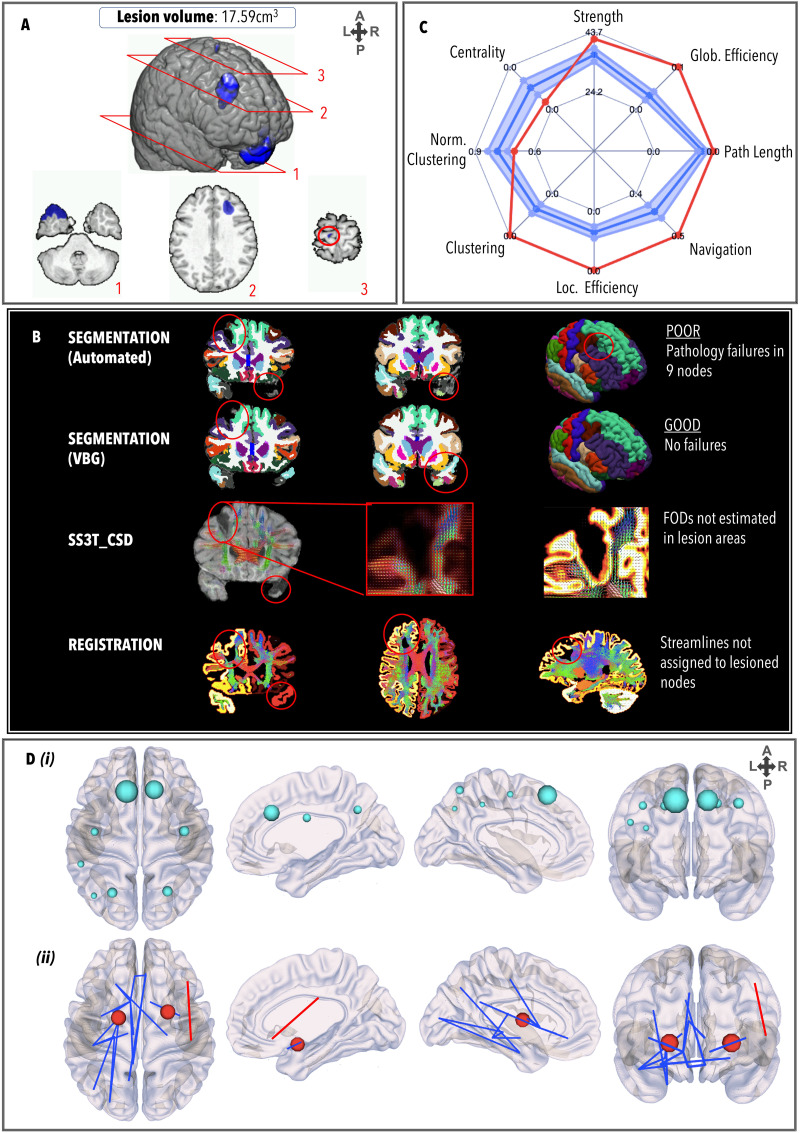
Same as Figure 3, for TBI4.

**Figure 6.**
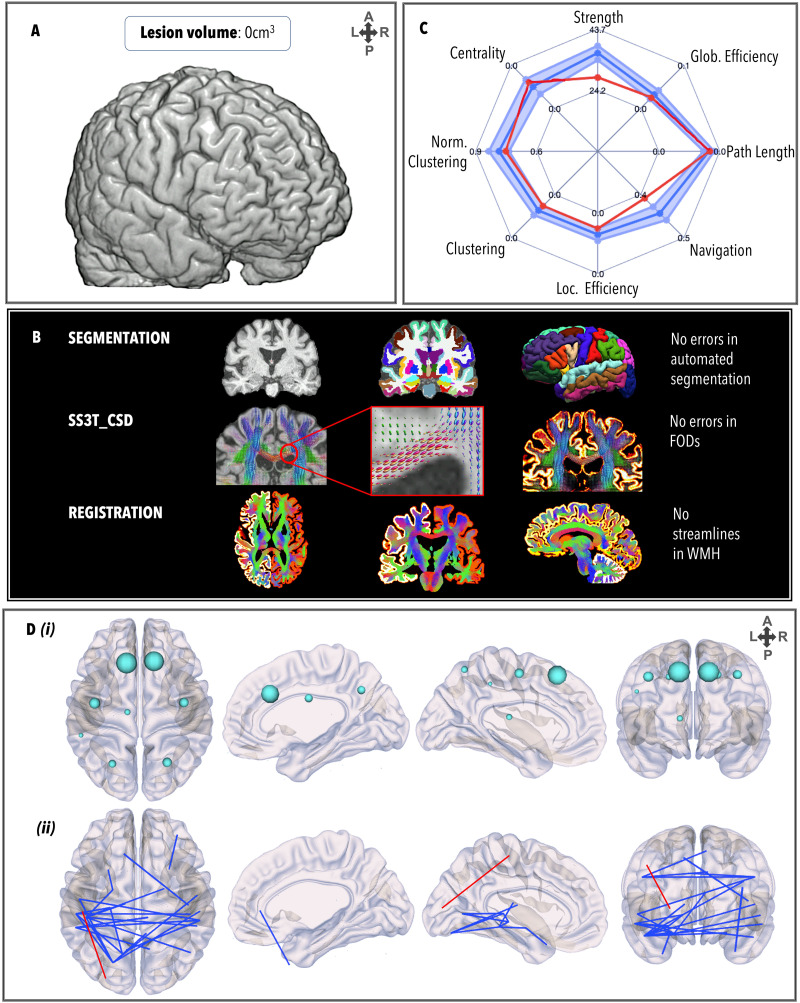
Same as Figure 3, for TBI5.

**Figure 7.**
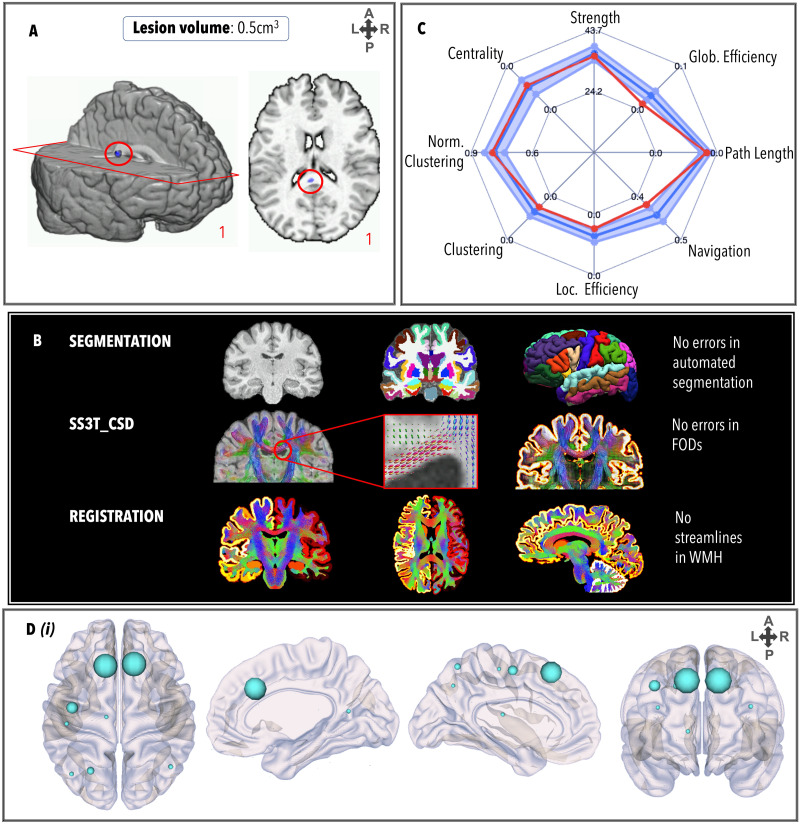
Same as Figure 3, for TBI6.

A key component of personalized connectomics is to localize network alterations in the brain relative to a healthy cohort. Nodal hubs and weakest edges were also examined for each individual patient based on comparison to the healthy controls. *Betweenness centrality* was used to identify brain regions essential for communication within the brain network (Freeman, 1978; Rubinov & Sporns, 2010), as done previously (Caeyenberghs, Leemans, Heitger, et al., 2012; Fagerholm, Hellyer, Scott, Leech, & Sharp, 2015; Raizman et al., 2020). The top 10% (*n* = 8) highest scoring nodes were identified as hubs; for the healthy control group these are shown in Figure 2, and for the TBI patients these are shown in Figures 3 to 7.

### Z-Score Matrix for Regional Analysis

An edge analysis scrutinized the white matter connections that drive overall differences in the network properties in greater detail (Wills & Meyer, 2020). A *z*-score matrix *Z*_*i*,*j*_ was derived, which describes the distance from the healthy control mean, divided by the healthy control standard deviation, between each subject’s connectivity matrix *T*_*i*,*j*_ and the controls *H*_*i*,*j*_ according to equations from a previous edgewise analysis (Wills & Meyer, 2020):

$$Z_{i,j}=\frac{T_{i,j}-\mu \left(H_{i,j}\right)}{\sigma \left(H_{i,j}\right)}.$$

Positive scores represent stronger edges in the TBI patient compared with controls, while negative scores represent weaker edges. False discovery rate correction (Benjamini & Yekutieli, 2001) was performed to determine which unique edges (of the upper triangle only, *n* = 3,528) were significantly different from the healthy control group. These edges are displayed on a glass brain. The same procedure was applied to examine how node strength in TBI patients deviated from healthy controls. Node strengths were calculated as the sum of strengths at each node (Rubinov & Sporns, 2010) and converted to *z*-scores using the healthy control mean and standard deviation. Positive scores represent stronger node strength in the TBI patient compared with controls, while negative scores represent weaker strength.

## RESULTS

### TBI1

TBI1 (Figure 3) had a relatively small lesion load (0.75 cm^3^) spanning the posterior segment of the right superior frontal gyrus and right precentral gyrus, and a DAI grade of 0. Registration between structural and diffusion images was unaffected by this lesion. There were no failures in the FreeSurfer pipeline and there was no need for VBG. FODs were generated at the site of the lesion (see red arrow) but did not meet streamline criteria for ACT. The GraphMe plot indicated that TBI1 has slightly weaker integration than healthy controls, including infra-normal navigation efficiency, strength, and clustering coefficient. Four alterations in the hub arrangement for TBI1 were observed, whereby the left thalamus (BC_left_ = 814) and putamen (BC_left_ = 730), and the bilateral superior frontal (BC_left_ = 3,224; BC_right_ = 3,394), superior parietal, (BC_left_ = 1,546; BC_right_ = 1,810), and lateral occipital gyri (BC_left_ = 646; BC_right_ = 618) were hubs and the bilateral precentral gyri and right thalamus did not meet the hub threshold. Four nodes, the left (*z* = −3.41, *p* = 6.50*e*^−04^) and right (*z* = −3.44, *p* = 5.61*e*^−04^) precentral gyri and left (*z* = −3.79, *p* = 1.51*e*^−04^) and right (*z* = −3.79, *p* = 1.50*e*^−04^) superior frontal gyri, had significantly lower strength than the healthy controls, while strength of the left (*z* = 4.18, *p* = 2.85*e*^−05^) and right (*z* = 3.44, *p* = 2.585*e*^−04^) nucleus accumbens were significantly higher. Weaker edges (*n* = 46 out of a total of 3,528 edges) were observed projecting across frontal, parietal, temporal, and subcortical areas, in particular the edges between the left posterior cingulate cortex and the right frontal pole (*z* = −7.57, *p* = 3.79*e*^−14^); the left thalamus and the left temporal pole (*z* = −6.47, *p* = 9.71*e*^−11^); the left lateral orbitofrontal gyrus and the left temporal pole (*z* = −8.19, *p* = 2.22*e*^−16^); and the left medial frontal gyrus and left temporal pole (*z* = −6.59, *p* = 4.23*e*^−11^). One stronger edge was observed between the right superior temporal gyrus and the right temporal pole (*z* = 5.96, *p* = 2.43*e*^−09^).

### TBI3

TBI3 (Figure 4) had a relatively large lesion load (15.46 cm^3^) involving primarily frontal regions (predominantly on the left), white matter hyperintensities in the medial right parietal lobe and the corpus callosum, and a DAI grade of 2. Prior to VBG, 10 nodes failed the quality assessment: VBG repaired nine nodes for parcellation. Registration showed that streamlines were not assigned to lesioned nodes. The GraphMe plot demonstrated an infra-normal graph metric profile in all domains. Two hub alterations were observed, whereby the bilateral putamen (BC_left_ = 871; BC_right_ = 932) were hubs, and the bilateral precentral gyri were not. Two nodes, the right medial orbitofrontal gyrus (*z* = −3.76, *p* = 1.68*e*^−04^) and the right pars orbitalis (*z* = −3.71, *p* = 2.09*e*^−04^), had significantly lower strength than the healthy controls. Weaker edges (*n* = 64) projected across the whole brain, especially the right frontal regions, including between the left frontal pole and the right superior frontal gyrus (*z* = −6.69, *p* = 2.15*e*^−11^) and right putamen (*z* = −8.96, *p* < 1.00*e*^−20^); the right medial orbitofrontal gyrus and the left insula (*z* = −7.75, *p* = 9.32*e*^−15^); the right insula and the right nucleus accumbens (*z* = −7.89, *p* = 2.88*e*^−15^); the right frontal pole and the right superior frontal gyrus (*z* = −8.15, *p* = 4.44*e*^−16^); and the right pars orbitalis and the right lingual gyrus (*z* = −7.92, *p* = 2.22*e*^−15^) and right cuneus (*z* = −6.57, *p* = 4.88*e*^−11^). No stronger edges were observed.

### TBI4

TBI4 (Figure 5) had a relatively large lesion load (17.59 cm^3^) involving bilateral frontal lesions and right temporal lesions, and white matter hyperintensities in the medial right parietal lobe and the corpus callosum. However, the DAI grade was low (0/1). Prior to VBG, nine nodes failed the quality assessment. All lesions overlapping with these nodes were repaired by VBG. Alignment between VBG-repaired nodes and streamlines indicated that any aberrant streamlines generated in areas with oedema/hemorrhage were not assigned to lesioned nodes. This patient exhibited supra-normal graph metrics in all domains except normalized clustering coefficient and centrality (which were infra-normal). Four alterations in the hub arrangement were observed, whereby the bilateral putamen (BC_left_ = 2,246; BC_right_ = 1,550), left palladium (BC_left_ = 1,210), and left inferior parietal (BC_right_ = 902) were hubs, and the bilateral precentral gyri and thalamic regions were not hubs. No nodes had significantly lower strength than controls, but two nodes—the left pallidum (*z* = 6.51, *p* = 7.35*e*^−11^) and the right putamen (*z* = 4.09, *p* = 4.31*e*^−05^)—had significantly higher strength. Weaker edges (*n* = 18) projected across the left hemisphere, including between the entorhinal and lateral occipital gyri (*z* = −9.62, *p* < 1.00*e*^−20^); the nucleus accumbens and the posterior cingulate cortex (*z* = −6.96, *p* = 3.21*e*^−12^), insula (*z* = −5.71, *p* = 1.10*e*^−08^), and lateral orbitofrontal gyrus (*z* = −6.65, *p* = 1.58*e*^−08^); the inferior temporal gyrus and the hippocampus (*z* = −6.57, *p* = 1.38*e*^−08^) and the amygdala (*z* = −6.17, *p* = 6.42*e*^−10^); and inter-hemispherically between the medial orbitofrontal gyri (*z* = −6.45, *p* = 1.08*e*^−10^). In the right hemisphere, weaker edges projected between the right insula and right accumbens (*z* = −5.97, *p* = 2.32*e*^−09^). One stronger edge was observed between the pars triangularis and postcentral gyrus (*z* = 5.80, *p* = 6.65*e*^−09^).

### TBI5

TBI5 (Figure 6) had no MRI-discernible lesion load and a DAI grade of 0. Many weaker edges were observed relative to healthy controls that connected the parietal, temporal, and subcortical lobes. There were no failures in the FreeSurfer pipeline, and no manual edits were necessary. FODs were generated correctly and registration between segmentation and tractography was free of error. The GraphMe plot revealed infra-normal strength and navigation. Two alterations in hub arrangement were observed, whereby the bilateral putamina were hubs (BC_left_ = 1,182; BC_right_ = 1,110), whereas the bilateral thalami were not. No significant differences in node strength were observed. Weaker edges (*n* = 25) projected inter-hemispherically across parietal, temporal, and subcortical areas. Weaker edges (*n* = 25) mostly projected to/from the left subcortical areas, such as between the amygdala and the temporal pole (*z* = −6.08, *p* = 1.17*e*^−09^) and the inferior temporal gyrus (*z* = −8.28, *p* = 2.22*e*^−16^); the inferior temporal gyrus and the hippocampus (*z* = −6.04, *p* = 1.46*e*^−09^) and the thalamus (*z* = −6.62, *p* = 3.53*e*^−11^); and the left cerebellum and the left middle temporal gyrus (*z* = −5.23, *p* = 1.61*e*^−07^) and right superior temporal gyrus (*z* = −5.59, *p* = 2.21*e*^−08^). One stronger edge was observed between the left postcentral gyrus and the left lateral occipital gyrus (*z* = 5.77, *p* = 2.13*e*^−08^).

### TBI6

TBI6 (Figure 7) had a small lesion in the splenium of the corpus callosum (0.5 cm^3^), and a DAI grade of 2. There were no failures in the FreeSurfer pipeline, and no manual edits were necessary. FODs were generated at the site of the lesion but did not meet streamline criteria for ACT. The GraphMe plot showed infra-normal global efficiency and navigation efficiency. Three hub alterations were observed, whereby the right caudate nucleus (BC_right_ = 722), right hippocampus (BC_right_ = 606), and right inferior parietal gyrus (BC_right_ = 680) were hubs, and the bilateral precentral and right superior parietal regions were not hubs. No significant differences in node strength were observed. No edges were weaker or stronger than the healthy control connectome.

## DISCUSSION

For the first time, we showcase an implementation of personalized connectomics in chronic moderate to severe TBI patients. In the following sections we discuss the defining characteristics of our single-subject profiles and explore ways in which our approach can contribute to improving existing methods of personalized structural connectome analyses in TBI patients.

### Single-Subject Network Profiling Observations

Our observations highlight a major caveat to approaches that attempt to identify a single graph metric that can be used as an adequate and parsimonious descriptor of structural network alterations in TBI patients (Imms et al., 2019). In accordance with Lv et al. (2020), we observed that each TBI patient showed a unique *pattern* of graph metric alterations, regardless of lesion load. For example, while both TBI1 and TBI5 patients had small lesion loads, patient TBI1 had lower brain network integration and segregation measures, compared with nonsignificant deviation from the normal range for patient TBI5. By comparison, TBI3 and TBI4 both had much larger lesions, but patient TBI3’s brain network profile showed infra-normal integration and segregation measures, while patient TBI4’s brain network was supra-normal. Our results highlight the benefit of using a multivariate *profile* of graph metrics that reveal individual differences in brain network topology otherwise obscured by group-level analyses. Importantly, with the incorporation of individual edge and hub comparisons, the location of the lesion can be compared with edge deterioration in single patients.

The sensitivity of individual graph metrics to cognitive impairments is still an active area of research. For example, there is evidence that communication measures such as navigation efficiency and global efficiency are sensitive to different types of processing speed in healthy controls (Imms et al., 2021), and that global efficiency is related to working memory improvements in TBI patients (Caeyenberghs et al., 2014). Table 2 and our previous meta-analysis (Imms et al., 2019) provide further evidence for the link between different graph metrics and cognitive outcome/performance. Such evidence suggests that with further validation, there may be benefits from distinguishing between patients with, for example, poor network communicability versus low clustering or centrality. Subsequently, graph metrics would hold value as proxies for network disruption that is indicative of different types of cognitive impairment, and as such could be useful in determining the type of cognitive program required by individuals.

### Improving Methods for Personalized Connectomics

Advancing individual brain network profiling has the potential to inform neuroimaging-guided personalized rehabilitation programs by providing network-based summary statistics with prognostic capabilities (Dichter, Sikich, Song, Voyvodic, & Bodfish, 2012; Stoeckel et al., 2014; Wing, Tucker, Fong, & Allen, 2017). More precisely, our approach can help to assess network alternations in TBI patients in the following three ways. First, regional connectome maps can be used as profiles of patients’ brain network topographies, thereby providing clinicians with time-efficient visual summaries of network disruption, asymmetry, hub alterations, and overall reductions in strength. Second, by comparing an individual patient with a healthy control reference group, we can observe portions of brain networks that are topologically altered but correspond to brain regions beyond the site of initial injuries. Finally, the GraphMe plots can be used longitudinally to map how the brain undergoes progressive secondary damage, recovery, and/or functional reorganization over time (Meningher, Bernstein-Eliav, Rubovitch, Pick, & Tavor, 2020; Osmanlioglu, Alappatt, Parker, Kim, & Verma, 2019).

The current best practice methods for inclusion of TBI patients’ scans that fail the FreeSurfer segmentation because of the presence of gross pathology—lesion masking and manual editing (Siegel, Shulman, & Corbetta, 2017)—are time-consuming and have low inter-rater reliability (Beelen, Phan, Wouters, Ghesquière, & Vandermosten, 2020). By contrast, use of the semiautomated lesion inpainting program VBG reduces the burden imposed by having to manually delineate lesions and avoids the exclusion of cases with large focal lesions that fail segmentation (e.g., from FreeSurfer; Radwan et al., 2021). Furthermore, we observed that the SS3T-CSD model (Dhollander et al., 2020; Dhollander, Mito, Raffelt, & Connelly, 2019) was suitable for constructing connectomes in the presence of lesions in all our TBI patients. SS3T-CSD removes the contributions from gray matter and cerebrospinal fluid components to increase the specificity of FODs to the white matter, while avoiding overestimation into gray matter and cerebrospinal fluid signal from the lesioned area (Khan et al., 2020). Combined with ACT tractography (Smith et al., 2012), streamlines are not generated in lesioned areas (e.g., see TBI1, Figure 3, panel C), and therefore anatomically disconnected regions do not have to be removed from connectivity matrices. This allowed us to calculate graph metrics from connectivity matrices of the same dimensions as those extracted from the healthy controls.

Our recent work (Caeyenberghs et al., 2018) together with other findings (Irimia, Chambers, et al., 2012; Irimia, Wang, et al., 2012; Williams & Gordon, 2010) suggest that we should utilize objective neuroimaging measures together with cognitive measures to improve the efficiency of training (i.e., a neuroscience-guided training approach using integrated cognitive training programs). Specifically, we should derive different brain and cognitive metrics to quantify subject-specific changes and locate them relative to a reference cohort. This information can assist clinicians in tailoring treatment plans based on the unique connectome and cognitive profile of each patient to better suit the needs of TBI patients. For example, compared with a reference healthy cohort, a patient with reduced values of graph metrics, like strength, efficiency, or centrality, in the presence of deficits in planning performance (e.g., as measured through, for example, the Tower of London test) may be used by a clinician as the evidence base to justify, design, and deliver a working memory training program (e.g., BrainGames [Verhelst, Vander Linden, Vingerhoets, & Caeyenberghs, 2017]; or Cogmed, https://www.cogmed.com), for this patient to ameliorate recovery.

Current approaches rely largely on clinical expertise of physicians/neurologists; the availability of a quantitative biomarker of white matter disconnectivity in moderate to severe TBI patients would be beneficial to supporting their expertise. However, future studies need to characterize individual variability of the human brain and behavioral outcomes in healthy controls, as well as clinical populations (Scarpazza et al., 2020). Specifically, we need to develop healthy reference ranges using large data repositories (e.g., Human Connectome Project, https://www.humanconnectome.org/; or Enhancing Neuro Imaging Genetics through Meta Analysis, https://enigma.ini.usc.edu/) for network metrics of structural brain networks across the adult life span. A reference standard of brain health would enable automated brain health reports for clinicians to compare against patients, allowing for personalized treatment programs. For the clinical utility of our approach to be useful, site-specific control data will also be necessary, to overcome inter-scanner variability, which systematically alters graph metrics (Kurokawa et al., 2021).

### Limitations

The implementation of personalized connectomics requires extensive validation and assessment of test-retest reliability. However, our study provides an initial framework of this approach using five TBI patients and a small healthy control reference group (*N* = 12; Attyé et al., 2020). Given the small number of healthy controls available from this dataset, we are limited in our ability to match healthy controls to TBI patients, or to provide a normative healthy control cohort against which to confidently distinguish deviance from healthy variability. This paper is a demonstration of a design that enables such comparisons. In future, healthy control norms will be created that match requirements for statistical comparison. As with any normative analysis (e.g., neurocognitive assessments), this would require the creation of large healthy control norm groups (*N* > 100) that are stratified by age bracket, sex, and possibly level of education, against which an individual patient can be matched for assessment of clinically meaningful differences using techniques such as quartile regression (Bourke et al., 2022; Jolly et al., 2021; Lv et al., 2020). Personalized connectomics should also include a patient group as an additional reference cohort (*N* > 100), to help clinicians understand how a patient is evolving with reference not only to healthy controls but also to patients with the same condition. The current work is intended as a demonstration of a new framework to which analysis of the clinical significance of graph metric alterations can be applied, rather than as a deliverable diagnostic tool in its current state.

Furthermore, our study utilized only *T*_1_ images for lesion identification; in the future, other structural imaging modalities such as fluid attenuated inversion recovery (FLAIR) and susceptibility-weighted imaging (SWI) should also be used in accordance with best practice guidelines for lesion identification (Olsen et al., 2020). Despite multiple expert raters and use of an established procedure (Adams et al., 1982), DAI grading remains subjective and requires independent confirmation of reliability. There is no consensus on the definition of hubs in the literature. In the present study, hubs were defined on the basis of values of betweenness centrality (Caeyenberghs et al., 2010; Fagerholm et al., 2015; Freeman, 1978; Raizman et al., 2020; Rubinov & Sporns, 2010). Other studies have employed multiple metrics including centrality, shortest path length, and clustering to identify a brain region as hub, which has been shown to be more stable (van den Heuvel, Mandl, Stam, Kahn, & Hulshoff Pol, 2010). Finally, cognitive outcomes associated with graph measures are still being evaluated; this progress will be essential for providing clinically informative personalized connectomes (Imms et al., 2021).

Methodological choices in the processing pipeline of diffusion MRI data can impact the biological interpretability and results of structural connectivity (Jeurissen, Leemans, Tournier, Jones, & Sijbers, 2013; Jones, 2010). Thus, we applied a state-of-the art diffusion MRI sequence and processing pipeline in MRtrix to avoid biases that may result in false pathways. Specifically, we used (a) SS3T-CSD with fiber orientation distributions estimated in the gray matter, white matter, and cerebrospinal fluid (to avoid overestimating the volume of white matter in voxels containing both signal types; Jeurissen, Tournier, Dhollander, Connelly, & Sijbers, 2014); (b) ACT to accurately determine where streamlines should be generated (Smith et al., 2012); and (c) an advanced tractogram reconstruction SIFT2 technique to provide a more biologically accurate representation of streamline count (Smith et al., 2015a) with the potential for stronger clinical relationships (McColgan et al., 2018). SIFT2 is found to decrease intersubject variability and increase biological accuracy of the structural connectome (Smith, Calamante, et al., 2020; Smith, Raffelt, et al., 2020; Smith et al., 2015a).

## CONCLUSIONS

Our results emphasize the translational potential for single-subject network analyses in the study of brain injury. Profiling individual patients based on their unique presentation provides insights into brain network disruption that are otherwise obscured by group-level approaches. The GraphMe profiling provides a novel user-friendly framework for leveraging graph metrics to benefit the individual patient by characterizing network-level brain alterations with potential prognostic relevance. Implementation of such a framework with stratified healthy control norms, and further evidence of diagnostic/prognostic ability of graph metrics, would enable us to progress towards a personalized medicine approach. Alongside group-based comparisons of patients against controls, such individual-level assessment frameworks are essential for translating connectomics to evidence-based clinical practice.

## ACKNOWLEDGMENTS

We would like to thank Dr. Alex Burmester for his help with the hierarchical drift diffusion modeling; and Michael Kean and the radiographers at the Royal Children’s Hospital.

## SUPPORTING INFORMATION

Supporting information for this article is available at https://doi.org/10.1162/netn_a_00277.

## AUTHOR CONTRIBUTIONS

Phoebe Imms: Conceptualization; Data curation; Formal analysis; Investigation; Methodology; Software; Visualization; Writing – original draft; Writing – review & editing. Adam Clemente: Data curation; Investigation; Resources; Writing – review & editing. Evelyn Deutscher: Formal analysis; Writing – review & editing. Ahmed Radwan: Resources; Software; Writing – review & editing. Hamed Akhlaghi: Data curation; Resources; Writing – review & editing. Paul Beech: Formal analysis; Writing – review & editing. Peter H. Wilson: Conceptualization; Resources; Writing – review & editing. Andrei Irimia: Writing – review & editing. Govinda Poudel: Conceptualization; Data curation; Formal analysis; Methodology; Resources; Software; Supervision; Validation; Visualization; Writing – review & editing. Juan F. Domínguez Duque: Conceptualization; Data curation; Formal analysis; Methodology; Resources; Software; Supervision; Validation; Writing – review & editing. Karen Caeyenberghs: Conceptualization; Formal analysis; Funding acquisition; Investigation; Methodology; Project administration; Resources; Supervision; Validation; Writing – original draft; Writing – review & editing.

## FUNDING INFORMATION

Karen Caeyenberghs, Australian Catholic University Research Fund, Award ID: 902915. Govinda Poudel, Australian Catholic University Research Fund. Karen Caeyenberghs, National Health and Medical Research Council (https://dx.doi.org/10.13039/501100000925), Award ID: APP1143816. Andrei Irimia, National Institute of Health (NIH), Award ID: R01 NS 100973. Andrei Irimia, US Department of Defense (https://dx.doi.org/10.13039/100000005), Award ID: W81-XWH-1810413. Andrei Irimia, Hanson-Thorell Family Research Scholarship. Andrei Irimia, James J. and Sue Femino Foundation. Peter H. Wilson, Research Centre Scheme, Australian Catholic University.

## TECHNICAL TERMS

Traumatic brain injury (TBI): A large force to the head that causes damage to the gray and white matter tissues of the brain.
Graph metrics: Summary measures that characterize graphs based on nodes (vertices) and edges (connections).
Personalized connectomics: Individual brain network profiling of disease characteristics using connectomics, in comparison to a healthy (normative) cohort.
Virtual brain repair: Inpainting is used to mask lesions, which are then filled in with healthy tissue prior to anatomical segmentation and parcellation.
Diffuse axonal injury (DAI): Trauma-induced shearing of axonal bundles caused by acceleration/deceleration forces that shift and rotate the brain inside the bony skull.
Single-shell 3-tissue constrained spherical deconvolution (SS3T-CSD): Differential resolution of fiber orientation distributions in gray matter, white matter, and cerebrospinal fluid separately.
Anatomically constrained tractography: Facilitates seeding of streamlines from the gray/white matter boundary during tracking by integrating anatomical information extracted from additional *T*_1_-weighted MRI.
Spherical-deconvolution informed filtering of tractograms (SIFT2): Uses spherical deconvolution to make the weight of the streamlines proportional to the underlying fiber orientation distribution.
